# Biomechanics of chest wall injury: implications for surgical stabilization and failure prevention

**DOI:** 10.3389/fsurg.2026.1841068

**Published:** 2026-07-07

**Authors:** Vladislav Muldiiarov, Zachary M. Bauman

**Affiliations:** 1 St. Joseph’s Hospital and Medical Center, Norton Thoracic Institute, Phoenix, AZ, United States; 2 Division of Trauma, Emergency General Surgery and Critical Care Surgery, Department of Surgery, University of Nebraska Medical Center, Omaha, NE, United States

**Keywords:** chest wall injury, fixation failure, rib fractures, sternal fracture, surgical stabilization of rib fractures, thoracic biomechanics

## Abstract

Chest wall injury impairs ventilation not only through pain, but also through loss of thoracic cage stability and disruption of regional mechanics. As surgical stabilization of rib fractures continues to expand, variability in operative technique and construct design remains, while recurring complication patterns suggest that mismatch between fixation constructs and physiologic loading may represent an underrecognized and potentially preventable cause of failure. Drawing on clinical guidelines, experimental biomechanics, advanced imaging, and contemporary finite-element modeling, this review synthesizes the mechanical behavior of the thoracic cage across the ribs, costochondral junctions, and sternum, and translates these principles into surgical planning. We propose a mechanics-based framework that links specific instability patterns to functional impairment and informs construct selection, including operative approach, plate and screw strategy, bone quality considerations, and management of sternal and costal cartilage involvement. In addition, we summarize common modes of fixation failure and discuss practical strategies to reduce their occurrence.

## 1 Introduction

Chest wall trauma remains one of the most common and consequential problems in acute care surgery. Large clinical series and contemporary reviews consistently show that thoracic trauma accounts for a substantial proportion of trauma presentations, with outcomes worsening as injury burden increases, particularly in older adults and in patients with multiple rib fractures (1–3). Beyond the initial insult, one of the central clinical challenges is that many patients appear relatively stable at presentation but deteriorate over the subsequent 48–72 h as atelectasis, pneumonia, retained hemothorax, and respiratory failure emerge (4–6). This delayed decline underscores that chest wall injury is not solely a pain disorder, but also a disorder of thoracic mechanics.

From a biomechanical perspective, the thoracic cage functions as an integrated system in which the ribs, costal cartilages, sternum, spine, and respiratory musculature share load and coordinate ventilation. Traumatic injury disrupts this coordinated behavior. Rib fractures, costochondral disruption, and sternal instability can uncouple normal chest wall motion, create regional compliance mismatch, and convert efficient expansion into ineffective or paradoxical deformation (7, 8). The physiologic consequences are predictable: increased work of breathing, impaired cough and secretion clearance, and progressive pulmonary decline that may initially appear disproportionate to early imaging findings (7, 8).

Over the past two decades, surgical stabilization of rib fractures (SSRF) has evolved from a selective intervention into an increasingly important component of modern thoracic trauma care. Randomized trials and meta-analyses in flail chest have demonstrated reductions in ventilator days and pulmonary complications, while prospective multicenter data suggest that selected non-flail patterns, particularly those with multiple severely displaced fractures, may also benefit, especially with respect to pain control and pleural-space complications (9–11). At the same time, consensus statements and best-practice guidelines have become available, yet substantial heterogeneity persists in real-world practice, including patient selection, timing of surgery, operative approach, and construct design (12, 13). That heterogeneity is clinically relevant because fixation systems differ in stiffness, load transfer, load-to-failure characteristics, and patterns of failure differences that may contribute to hardware-related symptoms, nonunion, or construct failure when implant behavior does not adequately match physiologic loading conditions (14, 15).

This review examines a persistent and underdeveloped challenge in the management of chest wall injury: the integration of thoracic cage biomechanics into routine surgical planning. It synthesizes current understanding of the mechanical behavior of the ribs, costochondral junctions, and sternum, and proposes a practical framework that relates distinct instability patterns to functional impairment and to construct selection, including operative approach, plate and screw strategy, bone quality considerations, and management of costal cartilage and sternal involvement. The purpose is not to establish a new scoring system, but to strengthen contemporary SSRF decision-making by making it more mechanically informed, more internally consistent, and less prone to preventable failure.

## 2 Methods

A structured narrative literature review was performed through February 2026 using PubMed/MEDLINE, with additional citation tracking from relevant reviews, guidelines, and primary studies. The search combined MeSH terms including “Rib Fractures,” “Thoracic Injuries,” “Flail Chest,” “Fracture Fixation, Internal,” “Fracture Fixation, Intramedullary,” “Biomechanic,” “Finite Element Analysis,” “Rib Cage,” “Sternum,” “Costal Cartilage,” and “Sternocostal Joints,” together with free-text terms such as “surgical stabilization of rib fractures,” “SSRF,” “chest wall injury,” “rib plating,” “hardware failure,” “costochondral injury,” and “sternal fixation.” English-language studies were included if they addressed thoracic cage biomechanics, chest wall instability, fixation strategy, construct behavior, implant-related complications, or management of sternal and costochondral injury. Clinical studies, biomechanical experiments, cadaveric studies, imaging-based analyses, finite-element investigations, guidelines, and relevant reviews were eligible. Studies focused primarily on pediatric populations, non-traumatic chest wall deformity, isolated analgesia without biomechanical or fixation relevance, non-thoracic fixation, or unrelated cardiothoracic procedures were excluded.

## 3 System-level thoracic cage biomechanics relevant to fixation planning

The thoracic cage should be understood as an integrated osseocartilaginous system rather than as a series of isolated ribs. At the system level, it must reconcile two competing mechanical demands: sufficient compliance to permit efficient ventilation and sufficient stiffness to preserve thoracic geometry, protect intrathoracic organs, and share load with the thoracic spine. Experimental and review data consistently show that the intact rib cage contributes meaningfully to thoracic spinal stability across the principal loading planes, with its greatest stabilizing effect observed in axial rotation and a stronger contribution in the upper thorax (16). Under physiologic follower-load conditions, removal of the rib cage substantially increases thoracic range of motion, while pressure-based studies indicate that the rib cage also shares applied moment and reduces thoracic disc loading (17, 18).

Importantly, this stabilizing effect is not uniform across the thorax. Watkins et al. reported that the intact rib cage increased thoracic spine stability by 40% in flexion-extension, 35% in lateral bending, and 31% in axial rotation, whereas simulated sternal fracture reduced stability by 42%, 22%, and 15%, respectively (19). Brasiliense et al. further showed that different regions of the rib cage resist motion in distinct ways: the sternum and anterior rib cage contributed most during flexion-extension, the posterior rib cage during lateral bending, and stability during axial rotation was directly related to the proportion of ribs remaining intact, with intact ribs accounting on average for 78% of thoracic stability (20). Stepwise reduction experiments by Liebsch et al. refined this concept by demonstrating that median sternotomy alone increased thoracic range of motion by 11.9% in flexion-extension and 21.9% in axial rotation, while disruption of the anterior rib cage produced even greater destabilization (21). Collectively, these findings suggest that rib fixation planning should be informed not only by fracture number, but also by the specific component of the thoracic ring that has been disrupted.

Within this system, the sternum functions not simply as an anterior bone, but as a central load-transmitting element of the thoracic ring (22). Clinical observations support this biomechanical role. Morgenstern et al. found that unstable AO/OTA type B and C thoracic spine fractures were more common in polytrauma patients with concomitant sternal fracture than in matched controls without sternal injury, and that same-segment sternovertebral injuries were associated with more rotationally unstable type C patterns (22). These findings support the view that sternal disruption may signal more extensive ring-level instability than is apparent from isolated fracture counts alone.

At the rib level, local geometry is a major determinant of both native biomechanics and implant performance. Mohr et al. demonstrated that ribs 3 through 9 exhibit substantial longitudinal twist, ranging from 41° to 60°, together with changing shaft curvature and a widening medullary canal from posterior to anterior regions (23). Subsequent morphometric studies confirmed marked regional variation in cortical thickness and cross-sectional properties, as well as age-related cortical thinning that is more pronounced in females and in the mid-rib cutaneous and superior regions (24, 25). Murach et al. further showed that rib structural behavior is strongly explained by cross-sectional geometry, reinforcing the concept that local anatomy, rather than implant type alone, is a major determinant of fixation behavior and fracture susceptibility (26).

Costal cartilage is another key determinant of thoracic mechanics, particularly in the anterior chest wall, yet its role is often underappreciated. Available data indicate that human costal cartilage exhibits anisotropic, nonlinear, and age-dependent mechanical behavior, with bending properties further influenced by sex, rib level, and the degree of calcification (27, 28). The perichondrium also contributes substantially to structural integrity; its removal reduces peak anterior-posterior reaction forces by 35% to 58%, supporting the view that costal cartilage behaves as a composite structure rather than as a homogeneous material (29, 30).

**Figure 1:**
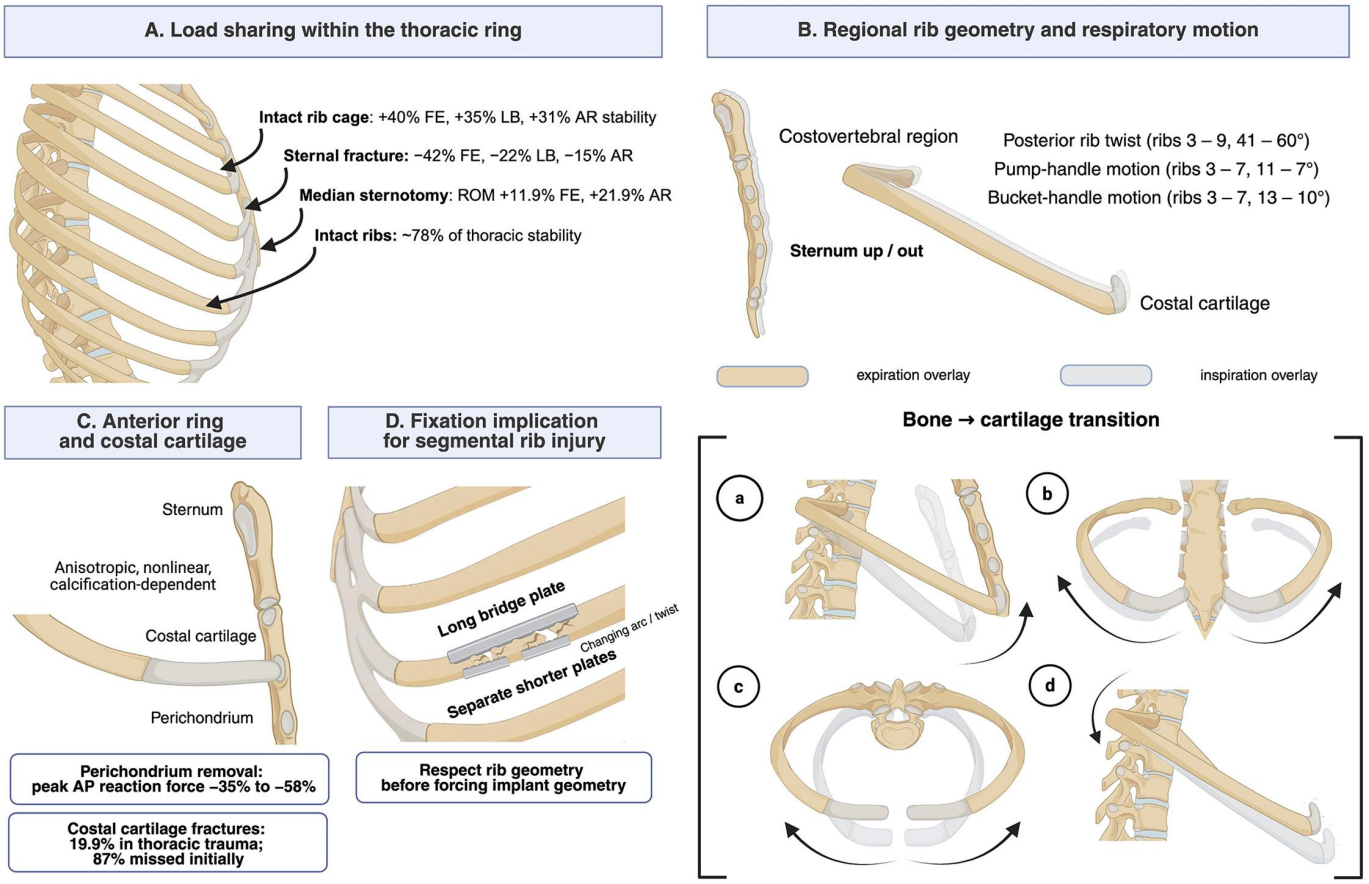
Thoracic ring load sharing, regional rib geometry, respiratory motion, and fixation implications. **(A)** The thoracic cage acts as a load-sharing ring in which the ribs, sternum, and costal cartilage together maintain stability and guide motion. Disruption of the anterior ring reduces stiffness and alters motion in flexion-extension (FE), lateral bending (LB) and axial rotation (AR). **(B)** Rib geometry and respiratory kinematics vary from the costovertebral region to the sternum. The posterior rib shows longitudinal twist, whereas the lateral and anterior segments undergo respiratory excursion. The lower-right subpanels illustrate the basic rib motions: (a) pump-handle, (b) bucket-handle, (c) caliper and (d) torsion. **(C)** The anterior thoracic ring comprises the sternum, costal cartilage, and perichondrium, each with distinct biomechanical properties. Costal cartilage is anisotropic, nonlinear and influenced by calcification. **(D)** These regional biomechanical features have direct implications for fixation of segmental rib injuries. Implant choice and plate configuration should respect rib curvature, twist and the transition from bone to cartilage.

Calcification further modifies costal cartilage mechanics and should not be regarded as a trivial imaging finding. Increasing calcification is associated with substantial increases in structural stiffness, while calcification pattern also influences behavior by increasing effective modulus and accentuating anisotropy when calcified regions remain contiguous with the rib (30, 31). Segment-level testing likewise demonstrates broad variation in bending, torsional, and tensile properties, consistent with the heterogeneous mechanical behavior of calcified cartilage (32). Extensive calcification also increases stiffness under both tensile and compressive loading, indicating that anterior chest wall compliance changes meaningfully with age and calcific remodeling (Figure 1) (33).

**Figure 2:**
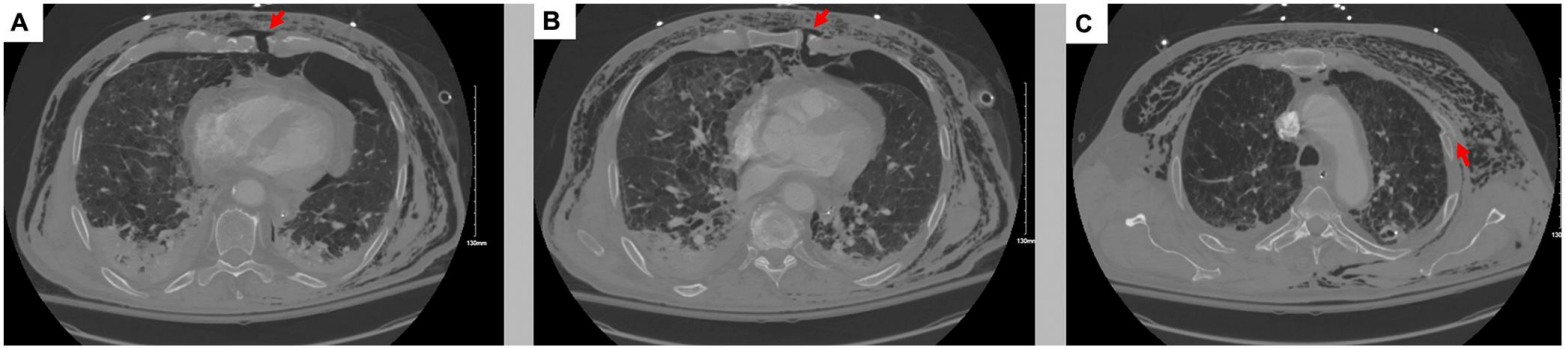
Concomitant rib and costal cartilage fractures. **(A,B)** Axial CT images show rib fractures with associated disruption of the anterior costal cartilage (red arrows). **(C)** Additional axial CT image demonstrates a rib fracture with adjacent subcutaneous emphysema (red arrow).

The clinical relevance of costal cartilage is heightened by the fact that these injuries are both common and frequently overlooked. Nummela et al. identified costal cartilage fractures in 7.8% of all whole-body trauma CT examinations and in 19.9% of patients with thoracic trauma, with the seventh costal cartilage most commonly involved (34). More recently, Forrester et al. reported that 14% of patients with radiologic chest wall injury had a costal cartilage fracture and that 87% of these injuries were missed on the initial trauma CT interpretation (35). Together with earlier imaging studies showing that cartilage fractures may be subtle on CT unless actively sought, these findings indicate that reliance on osseous rib fractures alone may underestimate the true extent of anterior ring disruption and mechanically relevant instability (Figure 2).

Rib-spine interactions add a further layer of regional heterogeneity. Oda et al. showed that the posterior elements, costovertebral joints, and rib cage each contribute importantly to thoracic stability and should be considered when thoracic instability is assessed (36). Duprey et al. quantified motion at the costovertebral joint and found a torsional range of motion of 16.9 ± 6.8°, substantially greater than that observed in the other tested directions, supporting the concept that these articulations permit controlled rib rotation while constraining other components of motion (37). More recently, Lebschy et al. directly measured stiffness in costovertebral articulations using rib segments that preserved adjacent vertebrae and ligamentous structures, reinforcing the view that these joints act as active mechanical stabilizers rather than passive attachments (38). Taken together with whole-cage studies, these findings help explain why posterior rib-spine dissociation and upper-thoracic true-rib injuries may generate disproportionately important rotational instability relative to their apparent displacement on imaging.

### 3.1 Biomechanics of instability in multiple rib fractures and flail segments

Chest wall instability after multiple rib fractures is best understood as a loss of coordinated load transfer and regional compliance rather than as a simple function of fracture count. In the intact thorax, rib excursion is level dependent. Wilson et al. demonstrated that ribs 3 through 7 rotate about an axis near the spine, with both pump-handle and bucket-handle components decreasing from 11° and 13° at rib 3 to 7° and 10° at rib 7, respectively (39). Subsequent finite-element work further showed that ribs do not behave as purely rigid bodies; instead, deformation of the ribs and, in particular, the costal cartilages contribute materially to thoracic motion and may augment the mechanical effect of the ventral intercostal musculature (40). The morphology of injury after blunt trauma is likewise region-specific. In a CT-based analysis of 380 cases comprising 3,735 rib fractures, Liebsch et al. identified a clear fracture hotspot involving ribs 4 through 7 in the lateral and posterolateral segments, indicating that instability most often develops in recurrent anatomic and mechanical zones rather than randomly across the rib cage (41).

In flail chest, the critical mechanical lesion is the creation of a disconnected thoracic segment that moves out of phase with the surrounding chest wall. Experimental canine studies showed that inspiratory displacement of fractured ribs is determined by the balance between pleural pressure-related inward forces and the outward action of the parasternal intercostals (42). Related work demonstrated increased inspiratory activity in the external intercostal and levator costae muscles within the disconnected segment, likely reflecting spindle-mediated responses to inspiratory muscle lengthening (43). Importantly, Shinozuka et al. argued that pendelluft is unlikely to be the dominant mechanism of respiratory dysfunction in this setting; instead, alveolar hypoventilation is more plausibly explained by wasted flail-segment motion that interferes with effective thoracic expansion (44).

A further distinction is that the radiographic morphology of a flail segment does not necessarily correspond to clinically significant paradoxical motion or respiratory insufficiency. Zierke et al. specifically noted that the current radiographic definition captures segmental fracture morphology, but not whether clinically relevant paradoxical breathing is actually present (45). In their model, increasing flail-segment size correlated strongly with lower tidal volume (R^2^ = 0.852, *p* = 0.003), greater absolute tidal-volume loss (R^2^ = 0.845, *p* = 0.0096), greater relative tidal-volume loss (R^2^ = 0.844, *p* = 0.0096), and greater compensatory respiratory work (R^2^ = 0.816, *p* = 0.0136). Tidal volume declined from 432 mL in the intact model to 324 mL in the largest injury configuration, corresponding to a 25.06% relative loss, while compensatory respiratory work rose to 33.44% in the same configuration (45). This distinction is consistent with serial CT data from Head et al., who showed that instability evolves over time: progressive offset was associated with greater fracture burden (median 7 vs. 4 ribs, *p* = 0.023), trended toward association with flail morphology (43% vs. 14%, *p* = 0.053), and occurred predominantly in the posterolateral region (46). Their neural-network analysis further identified ribs 4 through 6, posterolateral location, and multiple fractures within the same rib as the strongest predictors of progressive offset (46).

Postoperative imaging conveys a parallel message. Marasco et al. reported three-dimensional CT follow-up at 3 months after fixation strategies addressing only one fracture per rib of the flail segment found that, among patients with preoperative overlap or displacement, deformity often failed to improve despite stabilization of the lateral fracture site alone (47). The authors concluded that a one-fracture-per-rib strategy does not reliably prevent deformity, particularly when posterior fracture lines remain untreated (47). More recent evidence from Dong et al. reinforces this concern: delayed postoperative displacement occurred in 56.1% of patients, with posterior rib fractures representing the region most prone to secondary displacement (48).

Finite-element analysis has made this problem more quantifiable. In a pilot study, Bauman et al. found that loss of chest wall stability was greatest during axial rotation; a single rib fracture produced approximately 3% loss of chest wall stability, whereas complex fracture patterns reduced stability by more than 50%, with normalized interfragmentary motion increasing by as much as 230% (49). Notably, increasing the number of fractures in a non-flail pattern generated greater instability than a smaller three-rib flail configuration, challenging the notion that the binary label of flail chest alone adequately reflects mechanical severity. This concept was extended by subsequent work comparing complete and partial fixation. Complete stabilization restored chest wall behavior close to the intact state, whereas incomplete fixation left substantial residual instability. In a six-lateral-fracture model, partial fixation still resulted in up to 37% loss of chest wall stability; in lateral flail patterns, fixation limited to the anterolateral fractures left more than 40% residual instability, while motion at the untreated posterior-lateral fracture line increased further (50).

The link between mechanical instability and respiratory impairment is supported by both experimental and clinical evidence. In a full-thorax cadaveric negative-pressure breathing model, Slobogean et al. showed that a flail injury reduced inspiratory volume by 40 ± 19% and peak inspiratory flow by 35 ± 19%, both of which returned near baseline after fixation (51). In the randomized trial by Tanaka et al., surgical stabilization reduced ventilator duration (10.8 vs. 18.3 days), intensive care unit stay (16.5 vs. 26.8 days), and pneumonia incidence (24% vs. 77%), while improving percent forced vital capacity from 1 month onward (9).

Clinical follow-up studies further suggest that the functional consequences of instability depend not only on chest wall geometry, but also on the severity of displacement and the presence of associated parenchymal injury (52–54). Caragounis et al. found that postoperative CT-estimated lung volume increased from 3.51 L preoperatively to 5.59 L postoperatively and correlated strongly with both forced vital capacity (r_s = 0.75) and total lung capacity (r_s = 0.90) (52). Kishikawa et al. showed that in patients with flail chest and no surgery, pulmonary function recovered within 6 months in patients without pulmonary contusion even when residual chest wall deformity persisted, whereas patients with pulmonary contusion demonstrated sustained reductions in functional residual capacity and positional oxygenation abnormalities (53). More recently, Wang et al. reported that each additional severely displaced rib increased the odds of forced vital capacity below 80% at 3 months by 31%, and that the presence of three or more severely displaced ribs represented the optimal cutoff associated with impaired pulmonary ventilatory function (54).

Taken together, these findings suggest that clinically meaningful instability is shaped by fracture distribution, regional mechanics, and residual motion at untreated fracture sites, rather than by fracture count alone. This has direct implications for fixation planning: patterns involving multiple displaced ribs, posterolateral concentration, posterior residual fracture lines, or large disconnected segments are more likely to remain mechanically significant and may require construct strategies that extend beyond simple restoration of radiographic alignment.

### 3.2 Mechanics-based framework for SSRF planning

A mechanics-based approach to SSRF should begin with the pattern of instability rather than the number of fractured ribs alone. The clinically relevant question is which part of the thoracic ring has lost continuity: the anterior ring, including the sternum and costal cartilages; the lateral rib arc, where respiratory excursion and displacement are common; or the posterior rib-spine unit, where costovertebral and costotransverse attachments constrain motion and transmit load to the thoracic spine (16, 20, 22, 36–38). This distinction matters because each region fails differently and places different demands on fixation.

The next planning question is whether clinically meaningful residual motion will remain after a limited repair. This is particularly important in flail and segmental patterns, where stabilizing only the most accessible fracture line may restore local alignment but leave a second fracture line mechanically active. Contemporary WSES/CWIS guidance recommends stabilization of both fracture lines in flail or multiple-fracture series whenever possible, and stabilization of displaced ribs in non-flail patterns when technically feasible and clinically appropriate (8). These recommendations are consistent with postoperative imaging and finite-element data showing that partial fixation may leave residual instability, persistent deformity, or increased motion at untreated fracture sites (47–50).

The construct should then be matched to the expected loading environment. Unlike long bones, ribs cannot be immobilized after fixation; they remain exposed to repetitive respiratory, coughing, shoulder-girdle, and trunk-rotation loads. The goal is therefore not maximal rigidity, but restoration of enough continuity to reduce pathologic interfragmentary motion while preserving physiologic chest wall compliance (13–15, 55). This requires attention to fracture gap, working length, plate span, plate-bone contact, rib curvature and twist, local cortical thickness, and the presence of cartilage or sternal involvement (55–57).

No validated clinical threshold currently defines how much chest-wall or construct stiffness is required after SSRF. Therefore, mechanics-based planning should not be interpreted as a call for maximal rigidity. Its goal is to restore functional continuity, reduce pathologic motion at mechanically relevant fracture sites, and avoid residual gaps or stress concentration at mobile untreated fracture lines. This differs from long-bone fixation because ribs remain exposed to repetitive respiratory, coughing, shoulder-girdle, and trunk-rotation loads after surgery. In practical terms, preoperative planning should ask three questions: which fracture sites are mechanically relevant, which untreated sites may continue to move after fixation, and where will load be transferred once the construct is in place? This approach does not create a new indication for SSRF; rather, it provides a framework for construct planning once operative stabilization has been selected. The practical translation of these principles is summarized in Table 1.

**Table 1:** Mechanics-based translation of chest wall injury patterns into fixation planning.

Injury pattern	Biomechanical issue	Surgical planning implication	Evidence basis
Flail or segmental rib pattern	A free chest-wall segment may remain unstable if only one fracture line is stabilized.	When feasible, fixation should address both fracture lines that contribute to segment motion. A routine “one fracture per rib” strategy may be insufficient when the second fracture line remains displaced or mobile.	WSES/CWIS guidance, postoperative CT; finite-element studies. (8, 47–50)
Multiple displaced non-flail fractures	Significant mechanical dysfunction may occur without classic flail morphology, especially when several ribs are severely displaced.	Flail status alone should not determine fixation strategy. Once SSRF is selected, priority should be given to displaced fractures that alter chest-wall contour, rib length, or regional stability.	WSES/CWIS guidance; NONFLAIL trial; serial CT; pulmonary-function and finite-element data. (8, 11, 46, 49, 50, 54)
Posterolateral fracture cluster	Posterolateral fractures are exposed to substantial respiratory and rotational loading and may progress after partial fixation.	Posterior and posterolateral fracture lines should be reviewed deliberately on CT and 3D reconstruction. Fixation should be considered when safe exposure and reliable proximal fixation are achievable.	Rib morphometry; costovertebral biomechanics; serial CT; postoperative imaging; finite-element studies. (23, 36–38, 46–50)
Fracture near the transverse process	Short proximal rib length and deep paraspinal anatomy may limit secure fixation.	Posterior fixation should not be forced solely because the fracture is posterior. Fixation is most appropriate when adequate proximal purchase can be obtained safely.	WSES/CWIS guidance; technical and biomechanical literature. (8, 13, 36–38)
Anterior ring injury: sternum, costosternal joint, costochondral junction or costal cartilage	Anterior ring disruption may be missed if only osseous rib fractures are assessed; cartilage has variable stiffness and limited screw purchase.	The sternum and costal cartilages should be assessed routinely on CT. In selected unstable anterior injuries, sternum-to-rib spanning constructs or carefully planned costosternal, costochondral fixation may be considered.	Thoracic ring biomechanics; cartilage biomechanics; diagnostic CT studies; limited clinical series. (27–35, 56–60)
Poor bone stock, thin cortex, or complex rib curvature	Poor implant fit may increase screw pullout, plate lift-off or stress concentration.	Plate length, contouring, and screw strategy should be adapted to local rib anatomy. Separate shorter constructs may be preferable to a poorly contoured long plate.	Rib morphometry; fixation-system biomechanics; monocortical/bicortical fixation studies. (23–26, 55–57)
Large residual gap or short high-strain construct	Repetitive respiratory and cough loading can concentrate strain at the plate, screws, or untreated fracture line.	Rib length and alignment should be restored when possible. Residual gaps, short working spans, and stress risers adjacent to mobile fracture lines should be avoided. Gaps may need to be filled with a biological agent or bone autograft depending on its size if fracture end apposition is unable to be obtained.	General fixation principles; rib fixation biomechanics; hardware-failure literature. (13–15, 55, 56)

SSRF, surgical stabilization of rib fractures; CT, computed tomography; WSES, World Society of Emergency Surgery; CWIS, Chest Wall Injury Society.

### 3.3 Construct strategy and principles for durable fixation

Construct strategy should prioritize durable load sharing, adequate working length, and restoration of physiologic chest wall mechanics rather than focal over-rigidity (13, 55). Available rib fixation systems differ in stiffness, load transfer, and modes of failure, indicating that implant choice may not be functionally interchangeable (55). In contemporary SSRF practice, precontoured low-profile plates with locking fixation remain the dominant platform (8, 13). For segmental and flail patterns, longer plates are generally preferred because they distribute force across a greater working length and reduce stress concentration at the fracture site or at the ends of the construct (13). At the same time, this principle must be balanced against the marked change in rib curvature and twist along the rib arc (23, 56). In practice, a single long plate may be mechanically attractive yet technically difficult to contour accurately, particularly in posterolateral or multiply curved segments (23, 56). In these settings, separate fixation of the mechanically important fracture lines may be more practical and may provide a more reproducible construct than forcing a poorly matched long plate onto a complex rib surface. Shorter plates may also be reasonable when exposure limits long-segment fixation or when implant contouring would otherwise compromise plate-bone fit.

**Figure 3:**
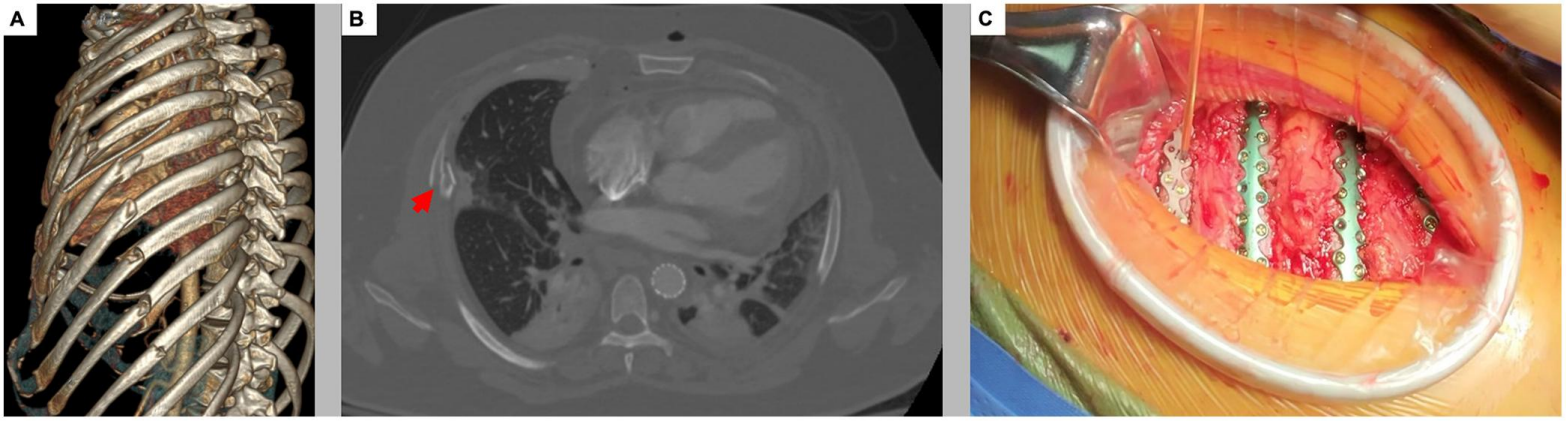
Rib fracture fixation with PEEK and titanium plates. **(A)** Three-dimensional CT reconstruction demonstrating multiple displaced rib fractures. **(B)** Axial CT image showing a displaced lateral rib fracture (red arrow). **(C)** Intraoperative view after surgical stabilization of the chest wall using PEEK and titanium plates.

Fixation durability depends not only on the plate itself but also on the quality of the rib-implant interface and the suitability of the platform for local anatomy (55). Modern SSRF systems include conventional extrathoracic plates, intrathoracic plates, polymer-based implants such as polyether ether ketone (PEEK) plates, and clip-based systems such as STRATOS (61–63). Intrathoracic plating may be advantageous in selected fractures where a minimally invasive or hybrid approach improves access, while early clinical data suggest that PEEK plates are a feasible alternative with better bioavailability, encouraging healing, and patient-reported outcomes (61–64). Clip-based systems may also be useful in anatomically challenging situations, although their role remains selective rather than routine. Rigid adherence to bicortical fixation is no longer necessary in modern SSRF (65, 66). Cadaveric data suggest that monocortical locking constructs can provide stability comparable to bicortical fixation (57). In practice, the more important issue is secure fixation matched to rib thickness, bone quality, fracture location, and implant design. Careful screw selection, plate apposition, and avoidance of implant mismatch are particularly important in thin ribs, posterior fractures, and osteoporotic bone (Figure 3).

Costochondral and sternal injuries require modification of standard rib-plating strategy rather than simple extension of routine osseous fixation principles. Current guidance supports spanning fixation from the sternum to the osseous rib in selected anterior chondral injuries, while recognizing that currently available systems were not specifically designed to achieve reliable screw purchase within cartilage (58, 59). However, small clinical series suggest that this approach to securing a plate in the costocartilage is feasible and safe and may yield satisfactory postoperative outcomes when constructs are carefully planned and contoured (58, 59). In a dedicated series of anterior chest wall and costal cartilage injuries, Varre et al. reported a low rate of hardware failure, supporting the view that such constructs can be durable in appropriately selected patients (60). By contrast, most isolated sternal fractures have traditionally been managed nonoperatively, with fixation generally reserved for unstable fractures, displacement, patients with respiratory compromise or other symptoms (i.e., uncontrolled pain, decreased upper extremity range of motion, etc), or symptomatic nonunion (67, 68).

Failure prevention begins with the same principle: avoid placing a construct into a mechanical environment it cannot tolerate. In their systematic review and meta-analysis, Choi et al. included 24 quantitative studies with 2,404 SSRF patients and reported a pooled hardware failure prevalence of 4% (3%–7%); mechanical failures were the most common type, and approximately 60% of patients with hardware failure underwent hardware removal (14). In the CWIS multicenter study, Sarani et al. reported hardware failure in 38 of 1,224 patients (3%); failure was asymptomatic in 40%, presented with pain in 42%, required explantation in 55%, and required repeat SSRF in only 10% (15). Thus, hardware failure is uncommon, but when it occurs it is usually mechanical and may be clinically relevant. Biologic complications such as infection appear less frequent, but when they occur, they may require reoperation and prolonged treatment (69).

Practical prevention includes CT-based review of the entire thoracic ring, restoration of rib length and alignment, avoidance of large residual gaps (hardware will eventually fatigue and break if gaps are left and the bone does not heal first), adequate working length and plate span, careful screw selection in thin or osteoporotic bone, and preservation of soft-tissue and periosteal blood supply. Durable SSRF is therefore less about selecting the stiffest implant and more about matching the construct to the patient's injury pattern, local anatomy, and expected loading environment.

### 3.4 Future directions

An important next step in chest wall surgery is to move beyond descriptive injury classification toward objective measures of mechanical instability. Current decision-making still relies largely on fracture number, displacement, radiographic pattern, and clinical deterioration, yet these variables do not fully capture the functional consequences of thoracic ring disruption. Future work should therefore focus on developing practical stability metrics that more directly reflect altered load transfer, regional compliance, and ventilatory impairment.

Patient-specific planning represents another important direction. Because chest wall mechanics vary according to rib level, curvature, cortical thickness, cartilage composition, and sternal involvement, injuries that appear similar on imaging may behave differently *in vivo*. Advances in CT-based morphometrics, three-dimensional reconstruction, and finite-element modeling may help support more individualized fixation strategies, including decisions regarding construct length, working span, screw configuration, type of plate (intrathoracic vs. extrathoracic, titanium plate vs. PEEK plate, or plates with screws vs. plates with superior and inferior rib clips) and the need to address anterior or posterior ring components. Modeling-informed surgical thresholds may also refine patient selection. Rather than reserving SSRF primarily for established flail chest or late clinical decline, future algorithms may identify instability patterns associated with increased respiratory work, progressive deformity, or elevated risk of construct overload. Although these approaches remain investigational, they may ultimately support more physiologically grounded and reproducible surgical planning.

## 4 Limitations

This review has several limitations. The available literature remains heterogeneous with respect to study design, patient selection, injury definitions, fixation techniques, and outcome reporting, which limits direct comparison across studies. In addition, much of the biomechanical evidence is derived from cadaveric, benchtop, imaging-based, or computational models that clarify mechanism but do not fully reproduce the physiology of the injured chest wall *in vivo*. For several issues central to fixation planning, including construct length, management of costochondral injury, and screw configuration, high-quality comparative clinical data remain limited. Important clinical variables are also incompletely represented in the current literature, including bone quality, regional thoracic stiffness, occult costochondral disruption, and patient-specific loading during coughing, mobilization, or rehabilitation. As a result, some practical recommendations are supported more strongly by biomechanical rationale and operative experience than by definitive prospective evidence. These limitations highlight the need for more standardized, mechanics-informed clinical research that links injury morphology, fixation strategy, and functional outcomes.

## 5 Conclusions

Chest wall injury should be understood not only as a pain syndrome, but also as a disorder of thoracic mechanics. Incorporating biomechanical principles into SSRF planning may improve construct design, reduce preventable failure, and support more consistent and physiologically appropriate fixation.
